# A chemical ecogenomics approach to understand the roles of secondary metabolites in fungal cereal pathogens

**DOI:** 10.3389/fmicb.2014.00640

**Published:** 2014-11-19

**Authors:** Yit-Heng Chooi, Peter S. Solomon

**Affiliations:** Plant Sciences Division, Research School of Biology, The Australian National UniversityCanberra, ACT, Australia

**Keywords:** fungal secondary metabolites, ecological genomics, chemical ecology, genome mining, plant pathogen

## Abstract

Secondary metabolites (SMs) are known to play important roles in the virulence and lifestyle of fungal plant pathogens. The increasing availability of fungal pathogen genome sequences and next-generation genomic tools have allowed us to survey the SM gene cluster inventory in individual fungi. Thus, there is immense opportunity for SM discovery in these plant pathogens. Comparative genomics and transcriptomics have been employed to obtain insights on the genetic features that enable fungal pathogens to adapt in individual ecological niches and to adopt the different pathogenic lifestyles. Here, we will discuss how we can use these tools to search for ecologically important SM gene clusters in fungi, using cereal pathogens as models. This ecological genomics approach, combined with genome mining and chemical ecology tools, is likely to advance our understanding of the natural functions of SMs and accelerate bioactive molecule discovery.

## INTRODUCTION

The interactions of fungal plant pathogens with their hosts are highly complex and involve both secondary metabolites (SMs) and small secreted proteins as pathogenicity factors (often defined as effectors). The role of SMs in mediating the virulence of fungal plant pathogens is increasingly being recognized (Mobius and Hertweck, 2009; Collemare and Lebrun, 2011). These fungal SMs facilitate infection by altering host cell structure or function via diverse mode of actions. Some of these SMs are host-selective toxins (HSTs) while others are non-host-selective general phytotoxins. Some classic examples of small molecule HSTs are found amongst the phytopathogens in the Dothideomycete class (Stergiopoulos et al., 2013; Muria-Gonzalez et al., 2014), including victorin, T-toxin, and HC-toxins. Other well-known examples of non-host selective phytotoxins include cercosporin, tentoxin, beticolin, depudecin, AAL-toxin, deoxynivalenol (DON) etc., each with different mode of actions.

The increasing number of phytopathogen genome sequences has revealed a large number of uncharacterized SM gene clusters in these fungi, particularly within the Dothideomycete but also other ascomycete pathogens such as *Magnaporthe* and *Fusarium* species. This indicates that we have merely scratched the surface of the SM repertoire in phytopathogens. Many SM gene clusters that encode the production of small molecules that have a role in host interactions are likely waiting to be discovered from these genomes. These phytopathogens SMs are equally likely to act as suppressors of other competing microbes that shared the ecological niches as well as deterrents of herbivores and fungivores (insects and other animals).

From a natural product discovery perspective, the flood of phytopathogen genome sequences presents an exciting opportunity for genome mining of bioactive molecules. Since eukaryotes, from fungi, plants to human beings, share many core biochemical pathways, many SMs that are targeted at plants and other eukaryotic microorganisms are likely to interact with macromolecules in human as well. Indeed, many molecules with human biological targets have been discovered from plant-associated fungi. Notable examples include, squalene synthase inhibitors zaragozic acids (Bergstrom et al., 1995), actin polymerization inhibitors cytochalasans (Scherlach et al., 2010), calmodulin inhibitors ophiobolins (Au et al., 2000), and various histone deacetylase inhibitors including depudecin, apicidin, and HC-toxin (Walton, 2006). These molecules have high clinical relevance and often serve as drug leads in drug discovery programs. Phytopathogens, which have to compete with other microbes in the environment, are also potential source of antimicrobial compounds. For example, aspergillomarasmine A, which was recently shown to be a promising metallo-β-lactamase inhibitor to combat antibiotic resistant bacteria (King et al., 2014), was originally isolated from the cereal pathogen *Pyrenophora teres* (Haenni et al., 1965). Despite that the biological targets of some of these bioactive SMs are known, the biological roles and ecological functions of these SMs often remain enigmatic.

**FIGURE 1:**
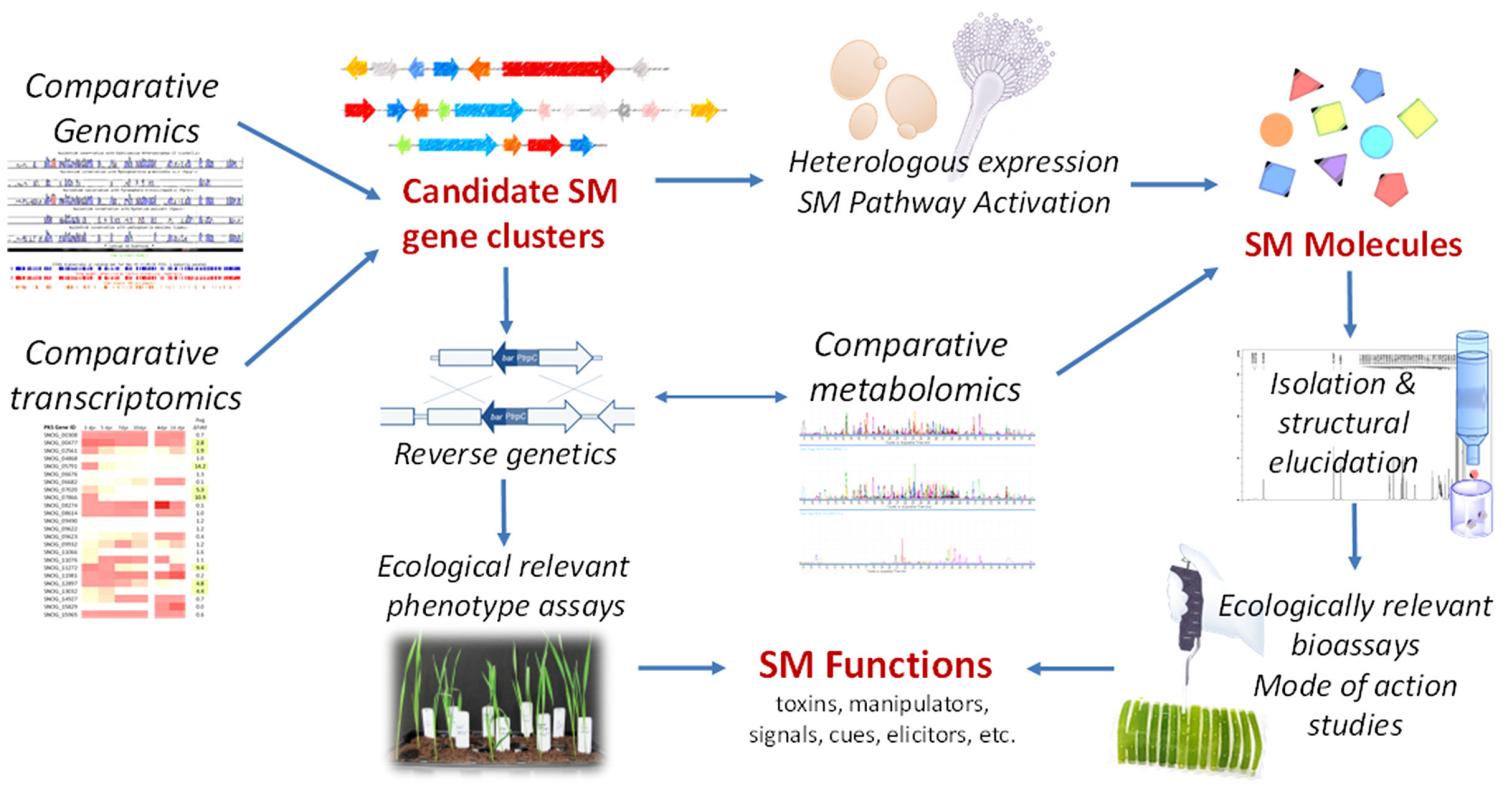
**An integrated chemical ecogenomic approach for understanding the functions of secondary metabolites (SMs) and bioactive molecule discovery.** The strategy incorporates the common tools in ecological genomics, genome mining and chemical ecology.

It has been recognized that the chemical ecology research can advance the discovery of bioactive molecules (Caporale, 1995). With the new next-generation genomic tools now at our disposal, we believe it is time to explore the synergy of ecological genomics and chemical ecology for advancing the understanding of the SM functions in phytopathogens. We termed this emerging integrated approach chemical ecogenomics. Combined with the increasingly mature genome mining tools and heterologous systems for expression of fungal SMs, this approach is likely to greatly accelerate the discovery of SM virulence factors and bioactive molecules. Similar strategies have been proposed for the study of insect pheromones (Tittiger, 2004), the role of fungal SMs in interactions with animals (Kempken and Rohlfs, 2010; Rohlfs and Churchill, 2011), and for natural product discovery in endophytic fungi (Kusari et al., 2012), coprophilous fungi (Bills et al., 2013) and Gammaproteobacteria (Vizcaino et al., 2014). Here, we would like to take some of these ideas one step further and to propose a basic chemical ecological framework for the study of SMs in phytopathogens, in particular, in cereal pathogens (**Figure 1**).

## CHEMICAL ECOGENOMICS – THE NEXUS OF CHEMICAL ECOLOGY AND ECOLOGICAL GENOMICS

Ecological genomics seeks to understand the function of genes and genome in biotic and abiotic interactions among organisms and their natural environments (Ungerer et al., 2008). Functional genomics tools, including comparative genomics, transcriptomics, and proteomics, are used to study the genome, transcriptome, and proteome dynamics in an ecological context. These studies often identify candidate genes that are important to a given ecological interactions or environmental niche. The identified genes are subjected to further functional verification. Similar studies focusing specifically on host–pathogen interactions in both human and plant pathogenic fungi are also often referred as pathogenomics (Pompe et al., 2005; Schmidt and Panstruga, 2011).

On the other hand, chemical ecology is the study of small molecules that mediate the biotic and abiotic interactions of organisms (Caporale, 1995). The small molecule metabolites that provide the organisms with adaptive advantages in specific ecological niches are often synonymous to SMs. A typical chemical ecology study often involves the isolation and structural characterization of the putative molecules involved in a given ecological interactions. The molecules are then used to test out the proposed function. The advantage of studying the function of genes and molecules in an ecological context is that they often provide important clues to their natural functions.

The SM biosynthetic genes that encode the production of SMs that mediate ecological interactions are essentially a subset of ecologically important genetic traits. Hence, studies in chemical ecology and ecological genomics have significant overlap and can be highly synergistic. Indeed, molecular genetics and genomics are also transforming the microbial SM (natural product) field, shifting the focus towards understanding biosynthesis and genes-to-molecules relationship (Walsh and Fischbach, 2010) and genomics-guided natural product discovery (Challis, 2008). Bringing these different, but related, fields together will help us bridge the gaps between genes, molecules, and functions.

The cereal pathosystems are perfect test beds for this multipronged chemical ecogenomics approach. Besides the large number of uncharacterized SM gene clusters in the genome, many of them are amenable to genetic manipulation and have established virulence assays (e.g., whole plant and detached leaf assays). An outline of how this emerging approach can be employed to study the SMs in cereal pathogens is presented in **Figure 1**. We will use some recent ecogenomics/pathogenomics studies in plant pathogens to illustrate the prospect of employing this strategy for understanding the SM functions in these pathogens and for bioactive molecule discovery.

## GENOME EVOLUTIONARY DYNAMICS OF SM BIOSYNTHETIC GENES IN FUNGAL PHYTOPATHOGENS

Secondary metabolite gene clusters are often not essential for the growth and survival of fungi under ideal conditions, but they confer selective advantages on the organisms by producing SMs that may act as defense molecules, signals, siderophores, or modulators in parasitic/endophytic interactions (Fox and Howlett, 2008; Collemare and Lebrun, 2011; Kusari et al., 2012). Thus, they are often subjected to adaptive evolution via a combination of genetic drifts and natural selection. Evolution mechanisms by gene loss, genetic mutation, gene duplication and divergence, genome rearrangement, fragment recombination and horizontal gene transfer (HGT) are commonly observed among SM gene clusters (Carbone et al., 2007; Patron et al., 2007; Fischbach et al., 2008; Proctor et al., 2009). The evolutionary dynamics of these SM gene clusters combined with the bio-ecological knowledge of the organisms can thus be used to infer the importance of individual SM gene clusters in environmental adaptations. Similar approaches have been recently employed to identify candidate effectors in plant pathogens (Stukenbrock et al., 2010; Gardiner et al., 2012; Ohm et al., 2012; Condon et al., 2013; Manning et al., 2013; Syme et al., 2013).

### INTRASPECIFIC COMPARATIVE GENOMICS – SPOT THE DIFFERENCES

**FIGURE 2:**
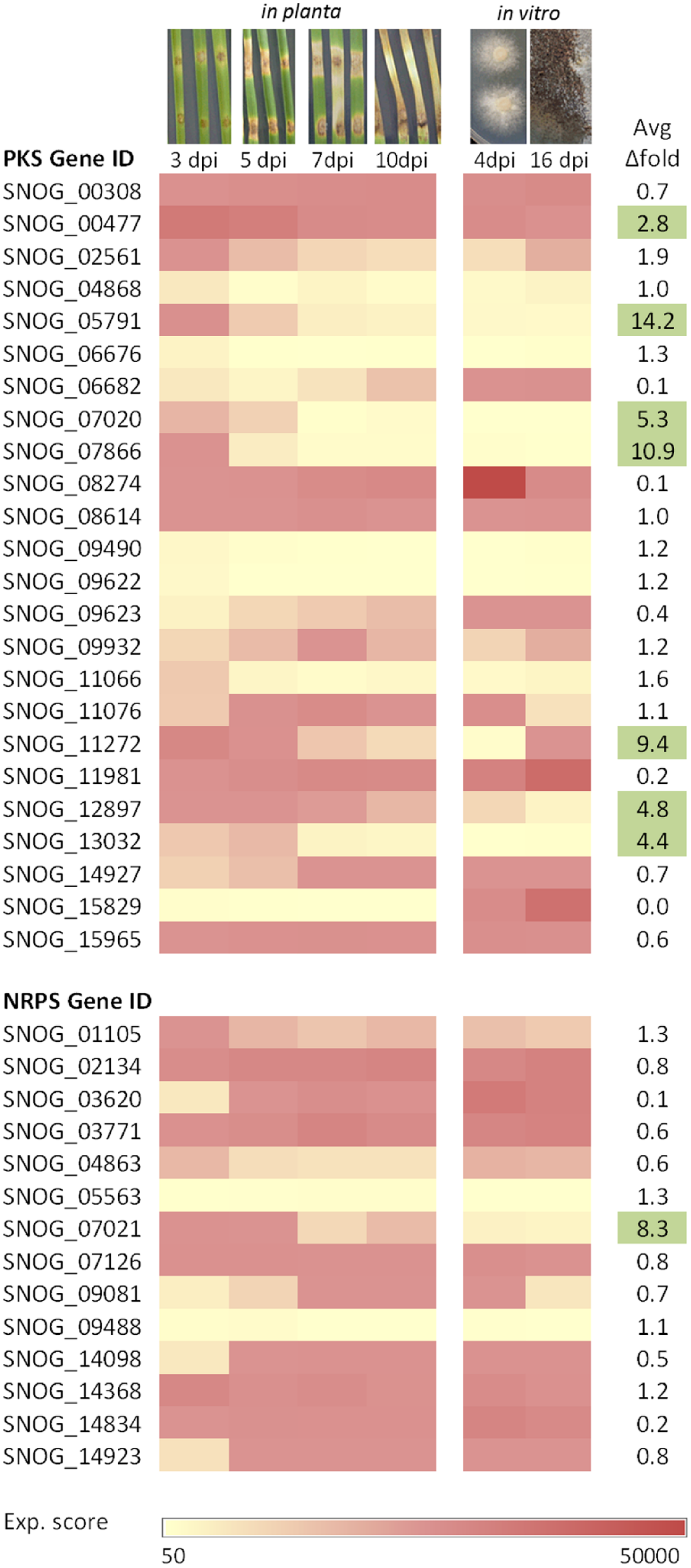
**Transcriptomic profiles of PKS and NRPS genes in *P. nodorum* on detached leaf assays (*in planta*) and during growth on minimal medium (*in vitro*).** The microarray data is based on Ipcho et al. (2012). Expression scores were normalized: <500 essentially indicative of no expression, >50000 indicates massive expression (out of dynamic range). Average fold difference is calculated by dividing the average expression score *in planta* over the average *in vitro* (highlighted in green indicates > two-fold difference).

With ready accesses to microbial genome sequencing enabled by next-generation sequencing technologies, genome sampling and re-sequencing is becoming a routine. In specific reference to cereal and other crop pathogens, comparative genomics and phylogenomics analyses on different strains of the same species with different host range or virulence profiles may reveal SM gene clusters that are important to virulence or pathogenicity. Using this approach, Brandon et al. have identified candidate SM gene clusters that may play a role in the virulence of *Cochliobolus* spp. (Condon et al., 2013). In one of the examples from the study, phylogenomic comparison of non-ribosomal peptide synthetase (NRPS) genes between pathotype 2 (isolate ND90Pr) and pathotype 0 (isolate ND93-1) of the barley pathogen *Cochliobolus sativus* revealed a NRPS gene cluster that is unique to pathotype 2. Deletion of one of the two NRPS genes in the gene cluster significantly reduced the virulence of *Cochliobolus sativus* pathotype 2 on barley cultivar Bowman. However, the SM molecule(s) encoded by this gene cluster remains to be identified. As a proof of concept, the authors further demonstrated the utility of this comparative phylogenomic approach to pull out the two polyketide synthase (PKS) genes previously identified to be involved in the host-specific T-toxin biosynthesis in *Cochliobolus heterostrophus* race T strains (Baker et al., 2006). As expected, the two PKS genes are present in all race T strains but absent in all race O strains (Condon et al., 2013). Comparative genomics of the wheat pathogen *Parastagonospora nodorum* SN15 strain with virulent and avirulent strains have also identified a significant number of strain-specific genes and genomic regions (Syme et al., 2013). Among those is a SM gene cluster that is absent in the genome of the avirulent SN79 strain but present in the two other virulent strains. Based on microarray data from a previous study (Ipcho et al., 2012), the gene cluster appeared to be up-regulated exclusively *in planta* during wheat leaf infection (**Figure 2**). Although it remains to be confirmed, it is tempting to speculate the gene cluster may encode a SM that plays a role in virulence.

### INTERSPECIFIC COMPARATIVE GENOMICS – SPOT THE SIMILARITIES

Horizontal gene transfer has now been recognized to be a common phenomenon among fungi (Fitzpatrick, 2012). In crop pathogens, HGT is thought to be a major evolutionary force that drives the emergence of new fungal crop diseases (Oliver and Solomon, 2008; Mehrabi et al., 2011). A well-known example is the interspecific horizontal transfer of the ToxA effector gene from *P. nodorum* to *Pyrenophora tritici-repentis*, resulting in the emergence of the tan-spot disease on wheat (Friesen et al., 2006). HGT of whole or partial SM gene clusters have also been proposed (Patron et al., 2007; Slot and Rokas, 2011; Wight et al., 2013). In a more extreme example, horizontal transfer of whole supernumerary chromosomes containing HST biosynthetic gene clusters has been proposed to confer pathogenicity to different pathotypes of *Alternaria* *alternata* (Akagi et al., 2009; Mehrabi et al., 2011). In fact, horizontal transfer has been proposed to be a principal driving force behind the evolution of clustering of SM biosynthetic genes (Walton, 2000). On the other hand, in some cases, the absence/presence of some SM gene clusters between closely related species can be explained by the loss of the gene clusters via genetic drifts (Chooi et al., 2010).

Given that the survival of a SM gene cluster is relying on its ability to confer advantages to the organism, we can expect that the conservation of a SM cluster across multiple species that share similar ecological niches may play similar bio-ecological roles. A good example is the recent discovery of immunosuppressive compounds from human pathogenic fungi (Chooi et al., 2013). Homology searches and comparative genomics identified a homologous gene cluster that is present among *Aspergillus fumigatus*, *Neosartorya fischeri,* and six dermatophytic fungi. The conserved gene clusters were demonstrated to produce the immunosuppressive compound neosartoricin (Chooi et al., 2013; Yin et al., 2013).

An example from the cereal pathogens is the SM gene cluster from *Cochliobolus carbonum* that encodes production of the host-selective HC-toxin required for pathogenicity to *hmhm* maize. Interspecific comparative genomic analysis identified HC-toxin gene cluster is present in another maize pathogen *Setosphaeria turcica* (Condon et al., 2013). Interestingly, the HC-toxin gene cluster is also present in another plant-associated fungus, *Alternaria jesenskae*, which does not appear to be pathogenic to most plants (Wight et al., 2013). Comparative phylogenomic analysis of NRPS genes among plant pathogens has also revealed that some NRPS genes have undergone recombination and modular rearrangement (Bushley and Turgeon, 2010). For example, modules 1 of ChNPS1 and ChNPS3 from *Cochliobolus heterostrophus* share high similarity to the modules in the AM-toxin synthetase of *Alternaria alternata* (Johnson et al., 2000), but other modules of ChNPS1 and ChNPS3 group with other cyclic peptide NRPSs and mono/bi-modular NRPSs in the phylogenetic analysis (Bushley and Turgeon, 2010). In such cases, care has to be taken in interpreting NRPS homology across different species as such NRPS module rearrangements will resulted in different SM products.

In *P. nodorum*, our detailed analysis of the SM gene clusters revealed several genes encoding PKSs and NRPSs that are highly conserved (≥70% protein identity) among several dothideomycete cereal pathogens, such as *Cochliobolus* spp., *Leptosphaeria maculans,* and *Pyrenophora tritici-repentis* (Chooi et al., 2014b). One common PKS gene among *P. nodorum, P. tritici-repentis,* and *L. maculans* has been shown to be responsible for the production of an antifungal compound phomenoic acid in *L. maculans* (Elliott et al., 2013). Phomenoic acid was proposed to be an antifungal substance used by the pathogens to outcompete other fungi in their environment.

## TRANSCRIPTOME DYNAMICS OF SM BIOSYNTHETIC GENES IN ECOLOGICAL INTERACTIONS

The production of SMs in fungi is highly regulated and often in response to specific biotic interactions and environmental perturbations (Keller et al., 2005; Brakhage, 2013). Therefore, the temporal and spatial expression of SM gene clusters may provide clues to the natural function of the encoded SM molecules in fungi. Traditionally, reverse transcriptase-PCR (RT-PCR) is used to monitor the expression of multiple backbone biosynthetic genes (e.g., PKS and NRPS genes) in fungi, while microarray allows the profiling of global transcriptome dynamics. Recent availability of next-generation RNA-Seq technologies has revolutionized transcriptomic profiling. Unlike microarray, RNA-Seq is not dependent on gene annotations and can provide information about transcript splicing as well. RNA-Seq also allows the simultaneous quantification of transcripts from more than one organism and is thus perfectly suited for the study of organismal interactions. For plant pathogens, these transcriptomic tools can be used to probe the expression of SM gene clusters during various stages of infection. Similar approaches can also be employed to probe fungal–fungal and fungal–bacterial interactions.

A classic example of SM gene cluster that is specifically expressed during host–fungus interactions is the ACE1 PKS-NRPS hybrid gene cluster from the rice pathogen *Magnaporthe grisea* (Collemare et al., 2008). The expression of ACE1 gene in *M. grisea* is highly up-regulated during the penetration into the host plant and the protein was localized to appressoria specifically. The gene *ace1* confers avirulence toward rice cultivar *Pi33* carrying a corresponding resistance (*R*) gene (Bohnert et al., 2004). ACE1 is likely to play a role in infection or manipulation of the host cell. Nonetheless, the identity of the SM product of ACE1 gene cluster and its function in *M. grisea* remain to be identified.

Recent global transcriptomic studies of plant pathogens have revealed several SM gene clusters that were expressed during infection. A remarkable example that highlighted the possible roles of fungal pathogen SMs *in planta* can be found in a recent RNA-Seq-based transcriptome study of *Colletotrichum higginsianum* (O’Connell et al., 2012). As many as 12 SM gene clusters (out of 39) were up-regulated before appressorial penetration and during biotrophic phase, but down-regulated during the necrotrophic stage. Similar observations were made on *Colletotrichum orbiculare* (Gan et al., 2013) and *Magnaporthe oryzae* (Soanes et al., 2012). Since the plant host remains healthy and asymptomatic during the biotrophic phase, the authors reasoned that the encoded SMs are unlikely to function as phytotoxins but perhaps as small molecule effectors that manipulate the host cells in ways that benefit the fungus or facilitate infection. Fungal SMs are often being screened for phytotoxic activities but their roles in biotrophy of fungi are largely unexplored and warrant further investigation.

To gain some insights in to the expression pattern of SM gene clusters in necrotrophic pathogens, here, we extracted the previous microarray data of *P. nodorum* during wheat leaf infection (Ipcho et al., 2012). During *in planta* stage, eight PKS genes and one NRPS genes, out of 24 and 14, respectively, were on average up-regulated twofold or more (**Figure 2**). There are also several genes that are up-regulated at the end of the necrotrophic phase (7 day post inoculation, dpi) before switching to saprotrophy. Close homologs of some of these genes can be found in other plant pathogens. For example, SNOG_05791, which was highly up-regulated at 3 dpi *in planta*, exhibits 82% head-to-tail protein identity to the alternapyrone synthase PKSN in *A. alternata* (Fujii et al., 2005). The final SM product and function of the PKSN gene cluster is yet to be characterized in *A. alternata* and it would be interesting to determine if the encoded metabolites play a role in the virulence of *P. nodorum* and *A. alternata*. We are in the process of teasing out the SMs encoded by these candidate gene clusters.

## BRIDGING THE GAPS BETWEEN GENES, MOLECULES AND FUNCTIONS

Ecological genomics is a powerful approach for inferring functionality and narrowing down ecologically relevant SM gene clusters. However, to obtain deeper insights into the bio-ecological functions of these SM gene clusters, the encoded SM molecules have to be first identified. Traditional chemical ecology studies often involved fractionation of an active crude extract guided by ecologically relevant bioassays (e.g., growth inhibition or behavioral response of an interacting organism). However, this method is not suitable if the compounds are only produced in response to specific biotic interactions. Modern metabolomic techniques have enabled the measurement of the metabolome dynamics of organisms during interactions (Prince and Pohnert, 2010). Nonetheless, in systems involved intimate physical interactions between two organisms (e.g., plant–fungal interactions), it is often difficult to track the origin of the metabolites. Furthermore, the amount of compounds that can be obtained through such interaction studies is often limited, which hinders further molecular characterization and mode of action studies.

Genome mining is increasingly becoming a popular approach for natural product discovery in recent years (Challis, 2008; Wiemann and Keller, 2014). To obtain the SMs from a candidate gene cluster, the SM pathway can either be expressed in various heterologous hosts after reconstructing the pathway with host-compatible regulatory elements or in the native producer via consecutive promoter replacements (Ahuja et al., 2012; Lim et al., 2012; Tsunematsu et al., 2013; Lazarus et al., 2014; Yaegashi et al., 2014). Alternatively, the expression of a silent SM gene cluster can be activated by overexpression of pathway-specific transcriptional regulator, if such regulator is present in the candidate SM gene cluster (Bergmann et al., 2007; Brakhage and Schroeckh, 2011). These methods are capable of producing the desired SM compounds in significant quantities and at the same time establish the important link between genes and molecules. The ready access to genome sequencing means that the availability of DNA sequences is no longer a bottleneck. However, translating SM gene clusters to small molecule products, via the above mentioned methods, remains a time-consuming endeavor. So far, genome mining efforts are focusing on (1) identifying the SM gene cluster of a known compound, (2) discovering analogous SMs from gene clusters that share homology to characterized ones, and (3) untargeted genome mining motivated by the quest to understand gene-to-molecule relationships. The application of ecological genomic tools adds a new dimension to genome mining and will help us navigate the vast genomic information in search for bioactive molecules (**Figure 1**).

Traditional reverse genetics and mutant phenotyping (i.e., virulence of mutants against the plant host) will still play important roles in casting light on the potential function of the SMs (**Figure 1**). For example, the presence and absence of specific compound in the wild type and mutant during inter-organism interactions can be verified by metabolic profiling. On the other hand, the information obtained from comparative phenotype assays between mutant and wild type will facilitate the development of specific bioassays to test the hypothetical function of the compounds obtained by ecogenomics-guided SM gene cluster mining. More recently, using this multi-pronged strategy, we have identified the SNOG_00477 PKS gene that is strongly expressed *in planta* to encode (*R*)-mellein production in *P. nodorum* by gene disruption and heterologous expression in yeast (Chooi et al., 2014a). We further demonstrated that mellein is a strong inhibitor of wheat seed germination. In conclusion, we believe this integrated chemical ecogenomic approach will accelerate the bridging of gaps between genes, molecules and functions, effectively linking genotype-to-phenotype. This multi-pronged approach is also applicable to other microbial ecological systems and will propel the next frontiers in fungal SM research.

## Conflict of Interest Statement

The authors declare that the research was conducted in the absence of any commercial or financial relationships that could be construed as a potential conflict of interest.
